# A typical case had rare immediate and delayed red man syndrome multiple times after norvancomycin injection: A case report

**DOI:** 10.1097/MD.0000000000032047

**Published:** 2022-11-25

**Authors:** Tianying Zang, Bingyang Liu, Lunkun Ma, Xiaojun Tang

**Affiliations:** a Department of Craniomaxillofacial Surgery, Plastic Surgery Hospital, Chinese Academy of Medical Sciences, Peking Union Medical College, Beijing, China.

**Keywords:** Anaphylaxis, case report, norvancomycin, Red man syndrome, vancomycin

## Abstract

Red man syndrome (RMS) is an adverse effect of vancomycin that usually occurs within minutes to tens of minutes after infusion. Previous literature reported that RMS rarely occurs again after the infusion speed is controlled.

**Methods:**

We report a case of immediate and delayed RMS that presented with fever, persistent lower extremity rash, shock, lymphadenopathy and pulmonary edema. This patient subsequently diagnosed with Sjögren's Syndrome, the time from NVCM infusion to RMS onset of this case ranged from 10 minutes to 54 hours, which are all rare in clinic and hard to distinguish severe RMS and IgE-mediated anaphylaxis.

**Results:**

After multidisciplinary consultation, the patient was diagnosed with RMS based on clinical manifestations and laboratory results. Patients' symptoms, signs, body temperature and disease progress were monitored, and an active search for causes was conducted. After a 20-day treatment, all the symptoms disappeared, the patient was transferred to immunology department to treat SS.

**Conclusion subsections:**

We reported a patient repeatedly developed fever and even shock when the infusion speed is normal, which was rare and similar as anaphylaxis. Therefore, the progression of RMS and its differentiation from allergy need to be further studied.

## 1. Introduction

A 34-year-old female was admitted to the hospital because of low eyebrow arch and underwent eyebrow prosthesis implantation. Due to prosthetic infection occurred after operation, norvancomycin (NVCM) was applied and then she had immediate and delayed Red man syndrome (RMS) multiple times. Her symptoms included fever, rash, muscle soreness, lymph node swelling and pain, even shock, and pulmonary edema. Multidisciplinary consultation made RMS diagnosis according to the clinical manifestations and previous history. After discontinuation of nonvancomycin, treated with glucocorticoids, symptomatic fluid infusion, and vasoactive drug pressure boosting, the patient was healed and transferred to the rheumatology and immunology department for further treatment.

A 34-year-old female accepted polytetrafluoroethylene prosthesis implantation in the brow arch region. The operation process was smooth and the patient did not complain of discomfort.

On the sixth day after prosthesis implantation, the patient found swelling and redness around the area of eyebrow arch that were progressively aggravated. Therefore, on the eighth day after implantation, the patient went to our hospital for emergency treatment of prosthesis removal, debridement and drainage, the pus was cultured for bacteria. Then the patient was hospitalized with the diagnosis “infection after prosthesis augmentation.” Positive pus cultures were obtained: staphylococcus aureus and serratia marcescens. So sensitive levofloxacin intravenous infusion was used, supplemented with negative pressure and abscess cavity flushing.

After admission, the patient’s temperature was normal, but the local symptoms were not relieved, so after consulting a pharmacist, we replace the antibiotics with NVCM intravenous infusion (0.8 g q12h) on POD5 after debridement (PODn refers to n days after debridement in this essay), and applied gentamicin, which is specifically sensitive to serratia marcescens, for local flushing. After 10 minutes infusion of NVCM, her neck began to turn red with no other symptoms of discomfort, and the red faded away after about 30 minutes in which process NVCM infusion continued.

**Figure 1.**
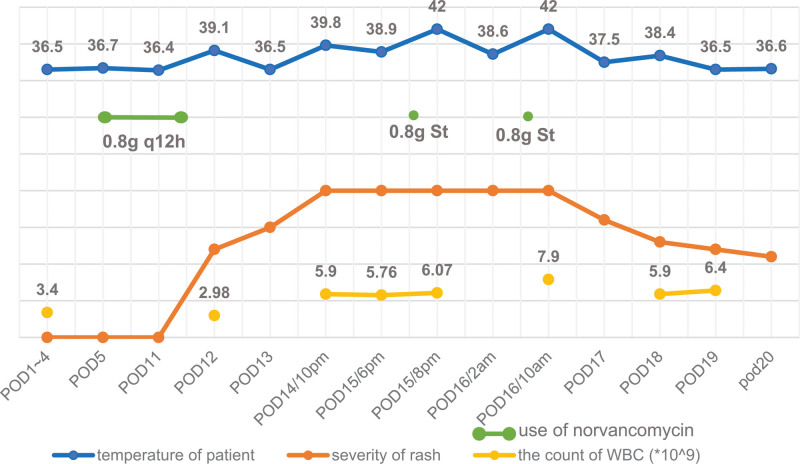
A chart that shows the relationship between the use of NVCM and body temperature, rash, count of WBC. (PODn: n days after debridement).

The local condition of brow arch had improved significantly until POD11. However, in the afternoon of POD12, after 5 hours of vancomycin infusion, she developed a fever of 39.1°C with no other discomfort. And when we took a closer look at her history, she had undergone allergy and urticaria in the past. complete blood count showed the white blood cell (WBC) count was 2.98 × 10^9^/L, which was considered related to the use of antibiotics, so NVCM was completely stopped, antipyretics was used and then her body temperature returned to normal (Fig. 1). In the evening of POD14, there was a sudden rise in her body temperature of 39.8°C, WBC count was 5.90 × 10^9^/L, and NEUT% was 68.0%. At the same time, she developed rash on both feet and lower legs, with flush on face, and then she was treated with antipyretics. Due to the persistent fever, on POD15, we further examined this patient and found she had newly developed bilateral submental and cervical lymphadenopathy and felt slight tenderness, CRP was 60.2, chest CT and abdominal ultrasound showed no abnormality. In the evening of POD15, she had fever of 38.9°C and chills again, without any trigger, complete blood count showed WBC count was 6.07 × 10^9^/L. After multidisciplinary discussion, we considered the possibility of sepsis, so loratadine and Calci D were given orally, norvancomycin was applied by intravenous infusion. Unfortunately, her body temperature increased up to 42°C about 30 minutes later, with the monitoring blood pressure (Bp) below 90/60 and continued to decrease, and there was no urine in past 6 hours.

**Figure 2.**
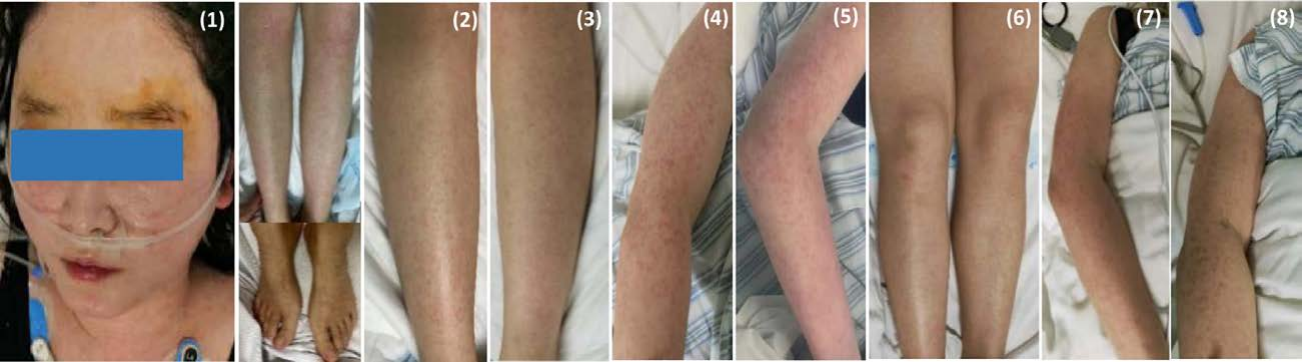
A photo of development of the rash in this patient: (1) The face, legs, and feet on POD14; (2) The right leg on POD15; (3) The left leg on POD15; (4) The right arm on POD15; (5) The left arm on POD15; (6) The legs on POD18; (7) The left arm on POD18; (8) The right arm on POD18.

**Figure 3.**
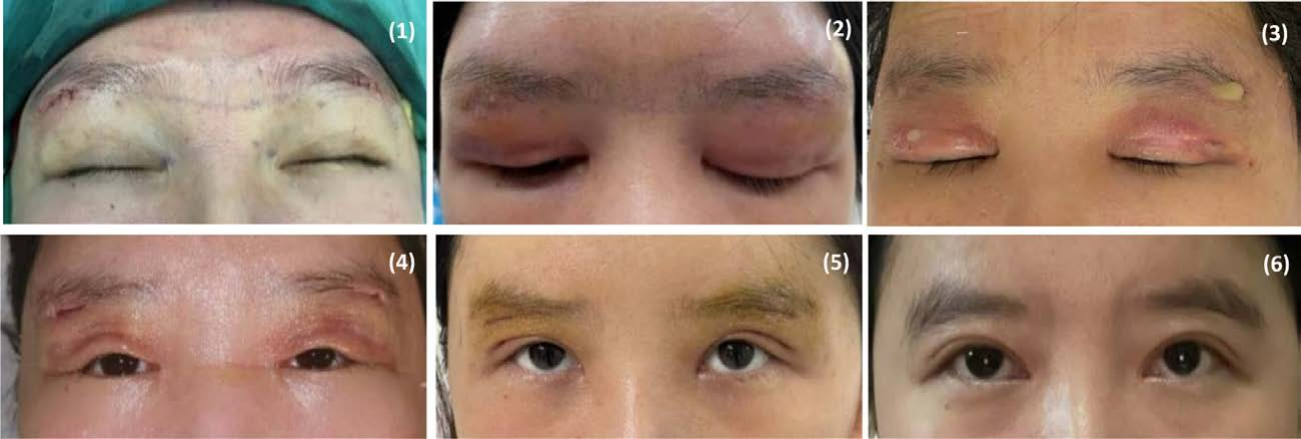
A photo of changes in the appearance of the surgical area over time: (1) Immediately after implantation; (2) The seventh day after implantation; (3) Immediately after removing prosthesis and debridement; (4) Day 10 after debridement; (5) Day 18 after debridement; (6) 3 months after debridement.

In order to avoid further deterioration, she was diagnosed as septic shock and transferred to intensive care unit (ICU) of a general hospital on POD16. Because of her uncontrollable body temperature, NVCM was given again and then she had a fever of 42°C, with rash got worse. Her chest CT showed diffuse interlobular septal thickening in both lungs and possible pulmonary edema. Then the ICU clinicians added imipenem, glucocorticoids, norepinephrine to maintain treatment. On POD 17, blood culture and metagenomics test report all indicated that this patient is unlikely to be infected. So, antibiotics were discontinued, glucocorticoids and supportive treatment was continued after multidisciplinary discussion, and concluded that RMS could be considered firstly (Fig. 2). At the same time, the ICU clinicians found her autoimmune related antibodies were abnormal: anti-SSA/Ro52 and anti-SSB antibodies were positive (+++), CD4/CD8 was 1.24, total B cells were 26–91%, IgM antibody was 55.9 Ru/mL, which means she had a combination of autoimmune disease. By the end of POD20, the patient did not have high fever again, with stable Bp, normal urine volume and the significantly relieved rash, and the brow arch region was basically healed. Subsequently, the patient was diagnosed as Sjögren syndrome and transferred to rheumatology and immunology department for professional treatment (Fig. 3).

## 2. Discussion

Vancomycin is one of the first choices for patients infected with β-lactam-resistant Gram-positive bacteria, which can cause hypersensitivity, kidney injury, and other side effects. Among them, hypersensitivity is a common and dangerous one that includes anaphylactoid reaction and anaphylactic reaction, it’s difficult to differentiate them according to clinical manifestations such as ever, flushing, rash, urticaria, pruritus to systemic erythema, and even hypotension.^[1]^ The former is RMS, that is more common, and caused by mast cells (MCs) degranulation under the direct drugs stimulation. There is a great difference in its incidence rate reported in the literature, but both clinical and research suggest that the incidence of healthy volunteers is much higher than that of patients, which is 30–90%, 3.7%–47% respectively,^[2]^ especially in men and black race.^[3]^ It is related to the route of administration, infusion speed and drug concentration.^[3]^ Anaphylactic reaction is an immune reaction mediated by immunoglobulin E, which is less common, independent of infusion rate, and insensitivity to antihistamines.^[4,5]^

The diagnosis of RMS mainly depends on the clinical manifestation and exclusionary diagnoses. There is no specific serological or histopathological diagnosis standard. RMS often appears within minutes of vancomycin infusion, or rarely appears several days after infusion. A study^[6]^ investigated the demographic data related to the side effects of vancomycin and found that among 18761 cases of hypersensitivity reactions caused by vancomycin, the most common hypersensitivity reactions were rash (32%) and RMS (16%). It is generally assumed that the occurrence of RMS is closely related to histamine release, but the presence of bacteria, malignant tumors, and diabetes can all affect the release of histamine.^[7]^ Therefore, bacterial infection consumes endogenous histamine, and vancomycin can stimulate histamine release only after the infection is controlled and the endogenous histamine is recovered. This explains the reason of delayed RMS and the reason why the incidence rate of RMS in healthy volunteers is higher than that in patients.^[8]^ In this case reported, this patient had flushing, rush, fever, and transient hypotension following NVCM infusion, she was thus diagnosed with RMS caused by NVCM after excluding other possible causes. At present, the existing detection methods for identifying high-risk people before vancomycin administration include^[4]^: skin prick test, intradermal skin test, basophil histamine release test, and drug stimulation test. However, a study conducted vancomycin skin test in 11 healthy people showed that skin test results could not predict RMS severity.^[9]^ At present, it’s certain that 50 mg/mL for skin prick testing and 0.005 mg/mL for intradermal skin testing nonirritating concentrations.^[10]^ Some scholars proposed that the severity of RMS is related to the amount of histamine released.^[6]^ Another scholar^[7]^ completed a randomized, double-blind, 2-way cross-over test on 10 adult males and concluded that there was no linear relationship between C-max of vancomycin and histamine release.

The differentiation of RMS and anaphylaxis is important but difficult in clinic. Tryptase level may help to distinguish,^[11]^ which is a serine protease produced and released by MCs. It starts to rise about 5 to 30 minutes after the event, reaches a peak after 1 to 3 hours, and returns to the basic value within 16 to 24 hours after the event.^[12]^ Some scholars^[13]^ put forward a consensus proposal on the basis of previous studies: when the acute serum tryptase (sAT) level is greater than [(1.2 * baseline serum tryptase (sBT)) + 2] µ g/L, it is of clinical significance to determine MC degranulation (sBT refers to 15 min to 3 hours after the onset of symptoms, sBT refers to before all symptoms appear or after remission for at least 24 h). Some scholars^[14]^ tested sBT of 57 patients with at least one allergic reaction history and found that the sBT level of patients with severe allergic reaction was higher than that of patients with mild and moderate reaction (the average level was 6.61 ng/mL and 4.71 ng/mL, respectively). Therefore, it was proposed that high sBT level might be helpful to identify patients with high risk of anaphylaxis. In this case, we did not complete these tests previously due to the lack of experience.

The prevention of RMS focuses on identifying high-risk people, controlling the infusion speed and concentration, and the controversial one: pretreatment of antihistamines. The first point, risk factors include^[15]^: white race, older than 2 years, previous RMS history, vancomycin dose greater than 10 mg/kg, vancomycin concentration greater than 5 mg/mL. This patient had urticaria and was allergic to cephalosporins. The second point is that some scholars have found that infusion of 1g/10mL vancomycin within 10 minutes can reduce systolic blood pressure by 25%–59% in 15% of volunteers.^[16]^ Therefore, slow administration can help reducing the incidence of hypotension, and it is generally recommended that the maximum concentration is 1 g/200 mL, and the infusion time should not be less than 1 hour.^[16]^ The third point is that some scholars^[17]^ compared the results of vancomycin injection and pretreatment (antihistamine or placebo) for 1 hour in 40 patients, they found injecting vancomycin within 2 hours reduced the incidence, severity of RMS and histamine concentration. Most scholars believe that the pre-use of H1 and H2 antihistamines can reduce the histamine mediated side effects caused by rapid vancomycin infusion.^[8]^ However, some scholars^[15]^ conducted a multicenter retrospective study on 546 subjects (0.5–21 years old) who received at least one dose of vancomycin intravenously, and concluded that previous use of antihistamine was a risk factor for RMS (*P* < .001).

For the treatment of RMS, the point is timely diagnosis, discontinuation of drugs, administration of anti-hypersensitivity treatment, and glucocorticoids, human immunoglobulin and continuous blood purification all can be used when necessary. Recent studies have pointed out that the activation of MRGPRX2 on MCs induces the release of several proinflammatory mediators, which include histamine, interleukins, tumor necrosis factor, and prostaglandin 2,^[18,19]^ vancomycin and staphylococcal delta toxin can activate MCs degranulation through MRGPRX 2, so the antagonists of MRGPRX 2 may alleviate RMS.^[20,21]^

In summary, this paper reports a case with allergic history, autoimmune disease, and complicated condition, who had RMS induced by multiple injections of NVCM. For this patient, we ignored her allergy history, we failed to make a timely diagnosis with experience, so we repeatedly applied vancomycin which caused the patient to develop RMS repeatedly. The diagnosis and treatment of RMS are difficult due to the lack of specific diagnostic methods, and existing skin tests often show negative results. Therefore, in the future, identifying the key points differential diagnosis and studying whether there is a linear relationship between blood histamine concentration and severity of RMS are important. In addition, whether the antagonists of MRGPRX 2 contribute to the remission of RMS needs to be further studied.

## Author contributions

**Supervision:** Xiaojun Tang.

**Validation:** Tianying Zang.

**Writing—original draft:** Bingyang Liu, Lunkun Ma, Tianying Zang.

**Writing—review and editing:** Xiaojun Tang.
